# Age effects in NHL draftees: a data-driven review of a multi-dimensional concept

**DOI:** 10.3389/fspor.2025.1593409

**Published:** 2025-09-30

**Authors:** Yiru Wang, Ben Csiernik, Kathryn Johnston, Joseph Baker

**Affiliations:** ^1^ Tanenbaum Institute of Science in Sport, University of Toronto, Toronto, ON, Canada; ^2^ Faculty of Health Science, Ontario Tech University, Oshawa, ON, Canada

**Keywords:** relative age effect (RAE), ice hockey, national hockey league (NHL), athlete development, player selection

## Abstract

**Introduction:**

Relative age effects (RAEs) have been extensively documented in youth sports, where artificial age cut-offs create advantages for relatively older athletes throughout development. Despite four decades of research, these effects persist in many sports, particularly in ice hockey, where misaligned cut-off dates between developmental systems and professional selection create unique challenges. This study examines varying age cut-off dates, athletes' development trajectories and career outcomes in elite ice hockey.

**Methods:**

Using one of the most comprehensive longitudinal datasets to date, the present paper also explores whether an “underdog effect” (i.e., where relatively younger athletes who survive selection barriers may achieve greater success) is present within the current sample of athletes. We analyzed the complete population of 10,485 NHL-drafted players spanning 44 years (1980-2024), examining birth quarter distributions, time to league entry, and career permanence (defined as playing ≥5 seasons and ≥268 games). Using Cox proportional hazards models and multinomial regression analyses, we investigated how birth quartile influenced player career trajectories while controlling for draft position, nationality, anthropometrics, and playing position.

**Results:**

Results revealed that while relatively younger players were significantly underrepresented in the draft, those who were drafted demonstrated superior career trajectories. In standard analyses, Q4 players showed a faster time to enter the NHL after getting drafted (HR = 1.32, 95% CI = 1.15-1.52), and Q3 players showed significantly higher likelihood of achieving permanence (HR = 1.39, 95% CI = 1.10-1.75). When accounting for the September 15 draft cut-off (2005-2024), a “dual disadvantage” was identified within the sample, with Q3 athletes showing the strongest effects, with 61% higher likelihood of achieving permanence (HR = 1.61, 95% CI = 1.27-2.05).

**Discussion:**

These findings support the “underdog hypothesis,” suggesting that relatively younger athletes who overcome systemic disadvantages develop compensatory skills that enhance long-term performance. Future athlete development systems should consider implementing strategies such as bio-banding or “future teams” to better support relatively younger athletes, potentially increasing talent retention across the entire player pool.

## 1 Introduction

The process of athlete development is complicated and complex; complicated in the sense that it involves many variables, across multiple domains (e.g., physical, psychological, cognitive, social, and environmental variables), and complex in the sense that these variables interact in a continuous, generally progressive way that advances over time. While researchers are only beginning to understand the web of interactions among these influences, those responsible for the delivery of sport to young athletes are tasked with developing strategies and policies to provide environments that are balanced and equitable for the greatest proportion of athletes. One of the ways in which sport systems have tried to do this is through the use of age-based standards for competition, training, and evaluation. In nearly all sports, it is the norm for athletes to compete against similarly aged peers throughout their development. On the face of it, this approach is reasonable and clearly intended to minimize differences between athletes as they age, mature, and learn. Moreover, it recognizes that the needs of athletes change as they develop (e.g., the requirements to keep a 7-year-old flourishing in a sport are different from those of a 17-year-old). To group athletes based on age, sports typically establish “cut-off” dates or “league ages” (i.e., setting a cut-off date of January 1 for an Under-13 [U13] team, means all athletes must be younger than 13 years as of Jan 1).

In the context of ice hockey, these age-based systems vary considerably depending on the country, and region within the country. In North America, and specifically in ice-hockey, athletes regularly participate in youth organizations until they reach high school, where diverging pathways begin (1). As an athlete progresses through the system, the available pathways become greatly impacted by location. For example, ice hockey players in Ontario, Canada will play at the under 16 AAA level in their age-16 season, leading up to the Ontario Hockey League draft. In contrast, an athlete born in Minnesota, USA, may primarily participate in Minnesota high school hockey at that same age. In both of these systems, there is an age cut-off which determines the minimum and maximum age for athletes to participate in. In Canada, the current standard for youth representative (i.e., rep league) hockey (which includes the highest levels a youth hockey player can participate in) includes athletes born between January 1st and December 31st of the same calendar year eligible to play on the same team.

Despite their prevalence, past research has emphasized several limitations associated with arbitrary cut-off dates as the standard for grouping individuals into cohorts for instruction and learning (2). Maturity-related biases, for example, reflect the tendency for differences in biological maturation between players in the same cohort/team to affect how players perform, and how they are evaluated (3, 4). These biases focus on how differences in rates of biological maturation between individuals affect performance on relevant tasks (e.g., early vs. later maturing players in assessments of speed, power, or endurance) (5).

Relative age biases, on the other hand, focus on how the cut-off dates used to group young athletes into age groups create artificial, but meaningful, divisions between the relatively oldest (those born nearest the date used for grouping) and the relatively youngest (born furthest from this date). The earliest relative age effects were identified in ice hockey by Grondin et al. (6) and Barnsley et al. (7), who found that relatively older players were more likely to be successful compared to relatively younger players. Since these initial studies, similar effects have been noted in many countries and several sports [see (2) for a review]. These effects have been impressively consistent over the past four decades [see (8, 9) for historical examinations of ice hockey and soccer, respectively] and difficult to eliminate (10). Moreover, what appears, on the surface, to be a relatively direct effect of an arbitrary date used for administrative purposes, is actually a multi-faceted series of effects [e.g., (11)] affecting different sports in nuanced ways [see (12, 13)].

Despite over 40 years of research in this area, most work has been rather straight-forward, providing breakdowns of birth rates in elite or highly skilled samples over 6-month (half years) or 3-month (quartiles) categories, compared to estimates of an expected distribution (normally an equal distribution of births across the year). Typical relative age effects show a decrease in the proportion of birthdates as the year progresses, highlighting the advantage of being relatively older in the group. However, maturity-related biases and traditional (i.e., within year) relative age effects are not the only age-related concerns in athlete development. For instance, constituent year effects examine differences between year cohorts in sports where youth are grouped into two-year age bands [e.g., ice hockey; (14)]. Other age-related factors have the potential to influence opportunities for success at different points across the athlete development pathway. For instance, when cut-off dates are not aligned across different systems or levels of competition, or when different policies use different cut-off dates (e.g., when the date used by one country differs from that used by another), age effects can be affected.

Despite these real and perceived advantages for athletes who are relatively older, relatively younger athletes who “survive” the system may also experience success. The phenomena, where athletes with a late birthday not only get selected to the system but survive and thrive, is known as the “underdog effect” (15–17). This effect reflects potential compensatory skills that relatively younger athletes must develop in order to compete and succeed against their relatively older peers, though the relationship between relative age and biological maturation is complex and not always aligned (16, 18, 19). In ice-hockey for example, Gibbs and colleagues (17) demonstrated support for the underdog effect, when examining National Hockey League (NHL) rosters of Canadian-born players from 2000–2009. Specifically, the authors found that a relative age effect was moderate for the average Canadian NHL player but reversed when examining the “most elite” professional players [i.e., All-Star and Olympic Team rosters; (17)]. Similarly, Kelly et al. (20) noted that later-maturing players in professional football academies were four times more likely to achieve senior professional status compared to their earlier-born counterparts.

In addition to categorizing the late maturing individuals as “underdogs,” Hill et al. (16) discussed “the released.” The released are those who struggle with overstimulation, may face injury or burnout, and do not “survive” the system (21, 22). Both of these phenomena may be especially relevant in North American ice hockey, where athletes may be “dually disadvantaged.” This term, adapted from Rubajczyk et al. (23), originally described athletes disadvantaged by multiple factors such as birth date and physical development. In the present context, we use it to describe athletes who face compounded disadvantages from misaligned cut-off dates: they are relatively young throughout youth development (with January 1 cut-offs) and then become the youngest eligible players in their NHL draft selection cohort (with September 15 cut-offs). For example, a player born in early September faces nearly a full year disadvantage in youth hockey and remains among the youngest when eligible for NHL selection.

One way to examine these influences, is to divide the year into four quarters (also called quartiles, i.e., four three-month blocks). This allows for the examination of differences between early, middle, and late quarters relative to a cut-off date. For example, with a cut-off date of January 1, quartile 1 (Q1) would span from January 1 – March 31, Q2 from April 1 – June 30, Q3 from July 1 – September 30, Q4 from October 1 – December 31. Athletes born in the third quartile of the year (between June and September) are younger relative to their age cohort throughout development but are also the youngest within their selection cohort for NHL, where athlete selection cohort cut-offs are currently between September 15 and September 14 of the next year. For example, an ice hockey player born on September 14 would be considered relatively young throughout their youth hockey development, and then further impacted by being the youngest player eligible for selection. At a population level, interestingly, between 2000–2019, the top three months for births in Canada (for those who responded to the survey) were each in Q3, with July being the month with the most births, followed by August, and September (24). While most sport systems recognize the importance of maturation, a deeper understanding of this bias could lead to more balanced long-term player development.

Given these unique challenges faced by relatively younger players in hockey, the present research seeks to explore whether *relatively younger NHL draftees show superior career trajectories despite systematic disadvantages compared to their relatively older peers.* By analyzing over four decades of NHL draft and career data, we examine whether relatively younger players who overcome selection barriers demonstrate different career trajectories, specifically, whether they enter the league faster and achieve greater career longevity than their relatively older counterparts. Additionally, we investigate how the misalignment between youth hockey and NHL draft eligibility cut-offs affects these patterns, providing a unique lens to understand the multidimensional nature of age effects in athlete development. Finally, we explore the concept of “dually disadvantaged” athletes, examining how those born in Q3 (July-early September) face compounded disadvantages: they remain relatively young throughout youth development with January 1 cutoffs, then continue as the youngest eligible players under the September 15 NHL draft cutoff, creating a unique natural experiment for understanding the “underdog effect” in athlete development.

Collectively, we aim to examine relative age effects in ice hockey from multiple angles, ranging from the consideration of unique developmental circumstances (e.g., do relatively older players enter the league quicker after selection than younger?) to the impact of incongruent cut-offs between developmental and professional systems on career outcomes (e.g., do Q3 athletes who face dual disadvantages achieve different permanence rates than other birth quartiles?)

## 2 Methods

### 2.1 Sample description

All athletes drafted to the NHL (*n* = 10,570 unique male athletes) between the 1980 NHL entry draft, and the 2023/24 NHL entry draft were retrieved from Elite Prospects (25), a third-party hockey data aggregator, and an NHL draft archive website (26). A total of 10,485 unique drafted players from the 1980/81 to 2023/24 NHL seasons were included in the analysis. The sample was comprised of forwards (*n* = 56.74%), defensemen (*n* = 33.27%), and goalies (*n* = 9.99%), with players predominantly from Canada (47.19%), the United States (22.99%), Sweden (7.70%) and Russia (7.12%).

### 2.2 Data collection and processing

Athlete information retrieval from these two data sources (i.e., the NHL website and Elite Prospects) was done using Python, primarily utilizing the request modules for data extraction and processing (27). From the Elite Prospects website, athlete draft selection information (i.e., overall draft pick, draft round) and season statistics (i.e., the seasons where an athlete played at least one game in, and total games played) were retrieved. From the NHL draft website, each athlete’s anthropometric information (i.e., height and weight) at the time of the draft selection was collected. Body mass index (BMI) was calculated using the athlete's height and weight information. Using an athlete’s selection year and selection number, data from both websites was merged to build the full dataset for our analyses.

In situations where players were selected more than once (*n* = 85 unique athletes), selection information from the athlete’s second time being drafted was used. The second draft was used under the assumption that the player had decided to re-enter the draft, making their second selection period more reflective of a team’s evaluation of them relative to their professional performance. Given that less than 0.01% of all players were selected more than once, we did not control for secondary selection in any of the analyses performed. Additionally, two players selected in the 1980s were removed due to missing information that could not be retrieved, resulting in a total of 87 selections being removed from the original dataset.

### 2.3 Variables

#### 2.3.1 Predictor of interest

**Birth quartiles:** To determine the relative age of each athlete, birth dates were separated into quartiles based on calendar year: January–March (Q1), April–June (Q2), July–September (Q3), October–December (Q4).

**Adjusted quartiles:** To account for the misalignment between youth hockey cut-off dates (January 1) and NHL draft eligibility cut-offs (September 15), we conducted a secondary analysis using adjusted quartiles. Q3 was redefined as July 1 to September 14, and Q4 as September 15 to December 31. This adjustment was particularly relevant for players drafted from 2005 onward (*n* = 4,080), following the 2005 NHL collective bargaining agreement.

#### 2.3.2 Outcome measures

Two primary career trajectory outcomes were defined: **Time to NHL Entry:** Number of seasons elapsed between draft and first NHL game appearance. Players who never entered were right-censored at the 2023/24 season, and **Time to NHL Permanence:** Number of seasons elapsed between draft and achieving “permanence,” defined as competing in ≥5 NHL seasons across ≥268 regular-season games. The five-season threshold was selected to align with peak performance windows in professional hockey, as prior research indicates NHL players typically reach peak performance between ages 24–26 (28). Given that the median time to NHL entry in our sample was 2 years post-draft, a five-year career would position players squarely within this peak performance window. The 268-game threshold represents the mean number of regular season games played by all NHL players in our dataset. Given the right-skewed distribution of games played, using the mean provides a more conservative threshold than the median, ensuring that “permanent” players have sustained regular participation rather than sporadic appearances across multiple seasons. This created three career outcome groups: “drafted but not entered,” “entered but not permanent,” and “permanent.”

#### 2.3.3 Control variables

Several covariates were included to isolate the effects of birth timing from confounding factors. Anthropometric variables (e.g., height, weight, BMI) were included to control for physical size differences at the time of draft, recognizing that body size may correlate with but does not directly measure biological maturation (19, 29). While these measures cannot capture the timing or tempo of biological development, controlling for physical size helps isolate birth quartile effects from size-related selection advantages. Player position was included given positional differences in developmental trajectories and selection criteria, with positions such as goaltending potentially less susceptible to the physical advantages (30). Nationality was included to help control for heterogeneity in development systems, as international variations in league structures, coaching philosophies, and age cut-offs create different selection environments that may moderate relative age effects (2).

#### 2.3.4 Statistical analysis

All statistical analyses were conducted in R using the survival and nnet packages (31–33). Statistical significance was set at *p* < 0.05, and 95% confidence intervals (CIs) were reported for all estimated parameters.

## 3 Results

### 3.1 Whole sample analysis

**Figure 1:**
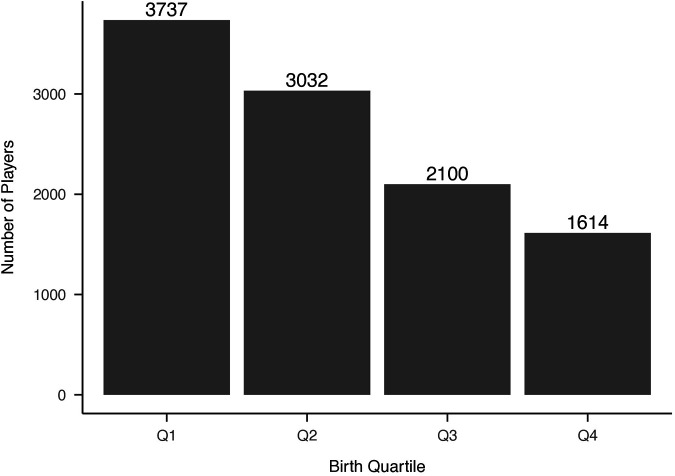
Distribution of players’ birth quartiles (unadjusted). Distribution of players’ birth quartiles, showing an overrepresentation of Q1 births, consistent with the relative age effect in ice hockey.

**Figure 2:**
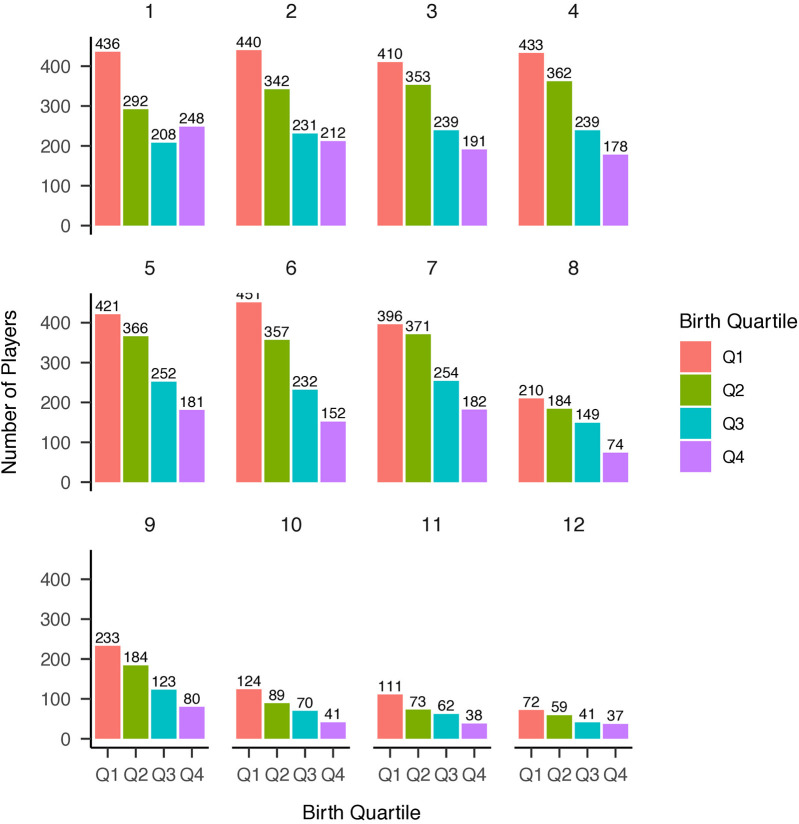
Distribution of players’ birth quartiles by draft round (unadjusted). Distribution of players’ birth quartiles by draft round, showing a persistent relative age effect across selection rounds.

**Figure 3:**
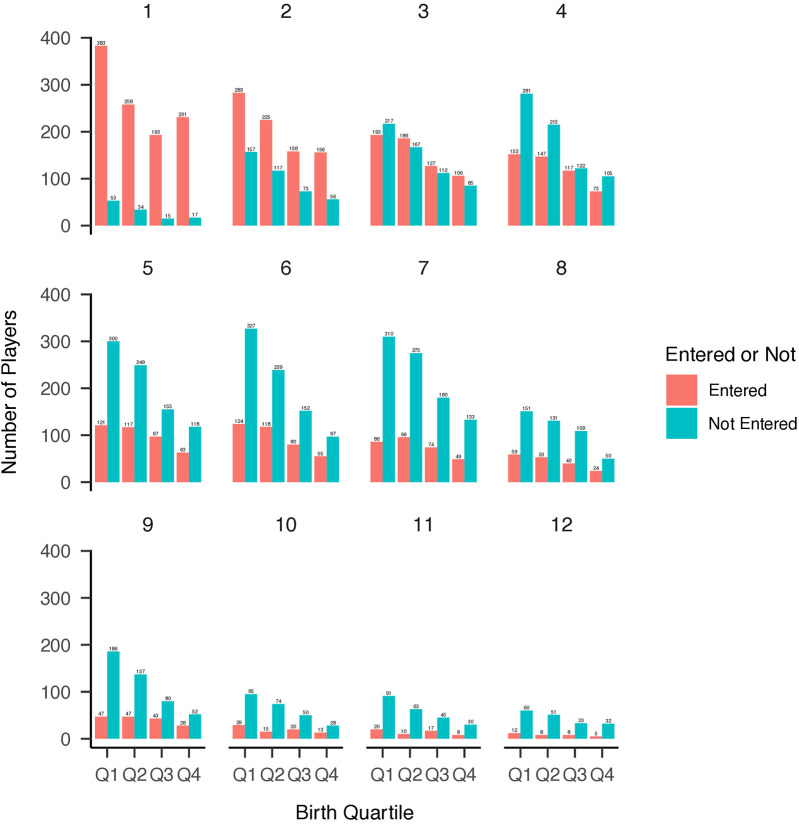
Distribution of birth quartiles by draft round and NHL entry status (unadjusted). Distribution of players’ birth quartiles by draft round, comparing those who entered the NHL versus those who did not. The relative age effect remains evident across both groups.

Distributions of NHL selections by birth quartile and NHL draft round, and birth quartile, NHL selection round, and NHL entrance status are reported in Figures 1–3 respectively. The average BMI at selection was 25.40 (SD = 1.75).

#### 3.1.1 Permanence status

Among all drafted players, 4,574 players (43.63%) successfully played at least one NHL game. However, only 1,757 players (16.76%) achieved permanence status. The median time to NHL entry was 2 years (IQR 1-4), while the median time to permanence from draft was 8 years (IQR 6-11).

#### 3.1.2 Time to NHL entry

**Figure 4:**
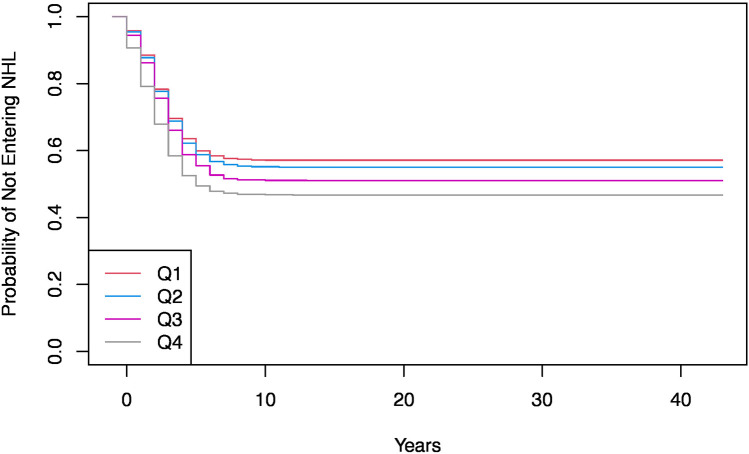
Kaplan-Meier curve for probability of not entering the NHL by birth quartile (unadjusted). Kaplan-Meier survival curves for NHL entry by birth quartile, showing the probability of not entering the league over time. Players born in Q1 exhibit a higher likelihood of entry compared to later quartiles.

Cox proportional hazards modeling demonstrated significant effects of birth quartile and draft pick on time to NHL entry (Figure 4). Later-born players exhibited faster entry times, with Q4 players reaching the NHL significantly earlier than Q1 players (HR = 1.32, 95% CI = 1.15–1.52, *p* < 0.001). However, when controlling for draft rank, the advantage diminished, with significant interaction effects between Q2 and Q3 players and draft rank (*p* = 0.015, *p* = 0.002, respectively). This suggests that while later-born players had greater likelihood of playing in the NHL, draft rank remains the strongest determinant of NHL entry timing.

#### 3.1.3 Time to NHL permanence

**Figure 5:**
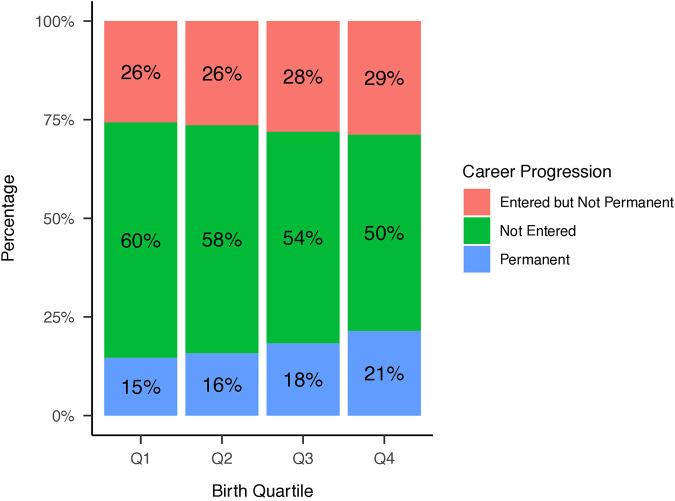
Career progression by birth quartile (unadjusted). Career progression percentages by birth quartile, illustrating differences in NHL entry and permanence. Q4 players have the highest rates of permanent NHL careers, consistent with underdog hypothesis.

The model assessing time to NHL permanence revealed that later-born players, particularly those in Q3 and Q4, were more likely to reach permanence status than athletes born in Q1 (Figure 5). Specifically, athletes in Q3 demonstrated a hazard ratio of 1.39 (95% CI = 1.10–1.75, *p* < 0.01), and athletes in Q4 demonstrated a hazard ratio of 1.25 (95% CI = 1.02–1.54, *p* < 0.03). Similar to time to entry, when draft position is accounted for, the strength of these relationships diminished with the hazard ratio for Q3 lowering to 1.00 (95% CI = 0.9976–1.0021, *p* = 0.90), and the hazard ratio for Q4 reduced to 1.001 (95% CI = 0.9984–1.0032, *p* = 0.50). A multinomial logistic regression was used as a sensitivity analysis, further supporting these findings. Players with lower draft ranks (higher pick numbers) were disproportionately affected by birth quartile: Q1 and Q2 players were significantly less likely to achieve permanence at lower draft ranks (*p* < 0.05), whereas Q4 players maintained a higher likelihood of permanence across all draft ranks.

### 3.2 Adjusted quartile analysis

**Figure 6:**
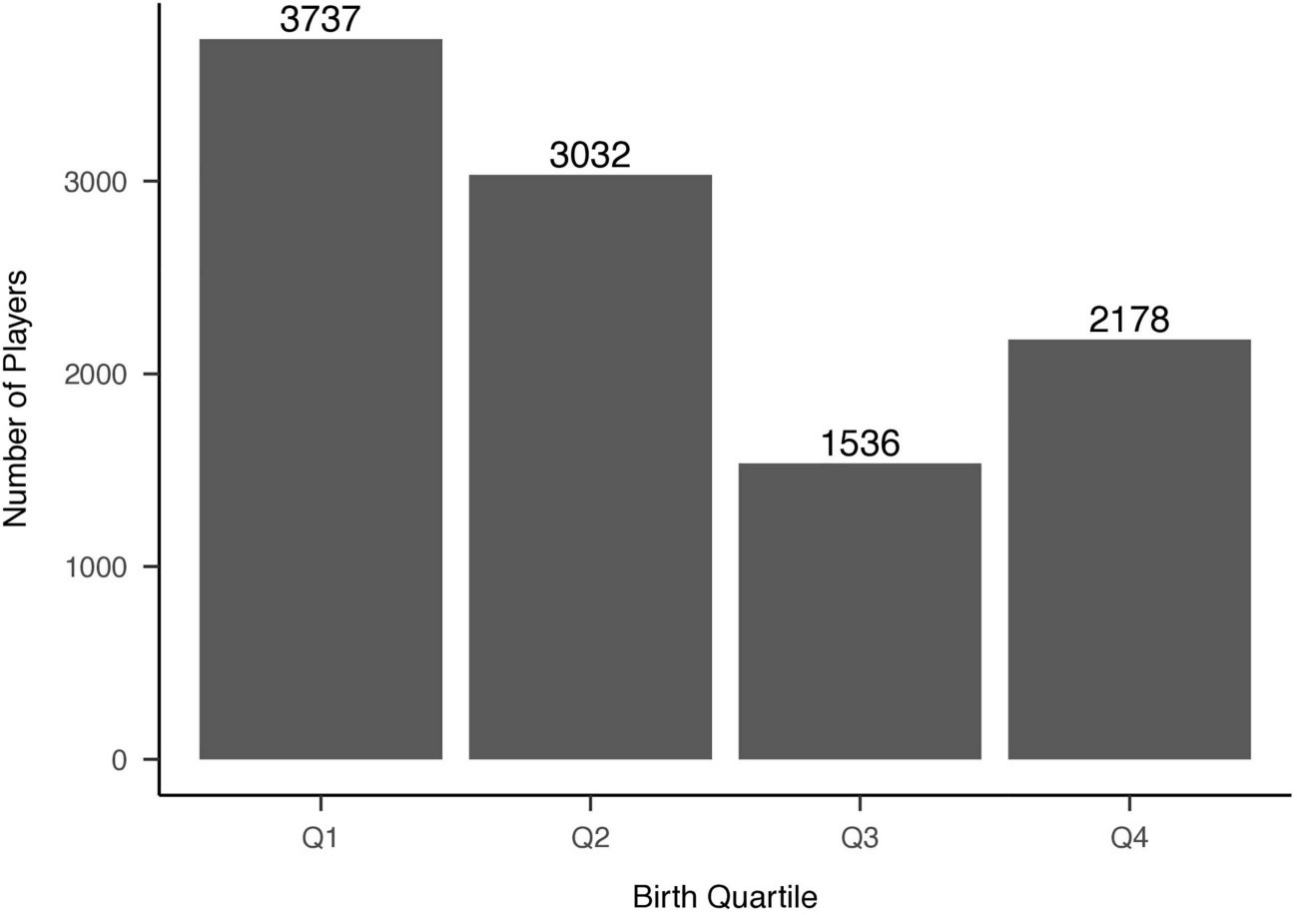
NHL selections from 1980–2024 using adjusted quartiles. Distribution of players’ birth quartiles using adjusted cut-offs (Q3: July 1 – September 14, Q4: September 15 – December 31) for NHL selections from 1980–2024. The relative age effect remains evident, though quartile distributions shift under the adjusted framework.

**Figure 7:**
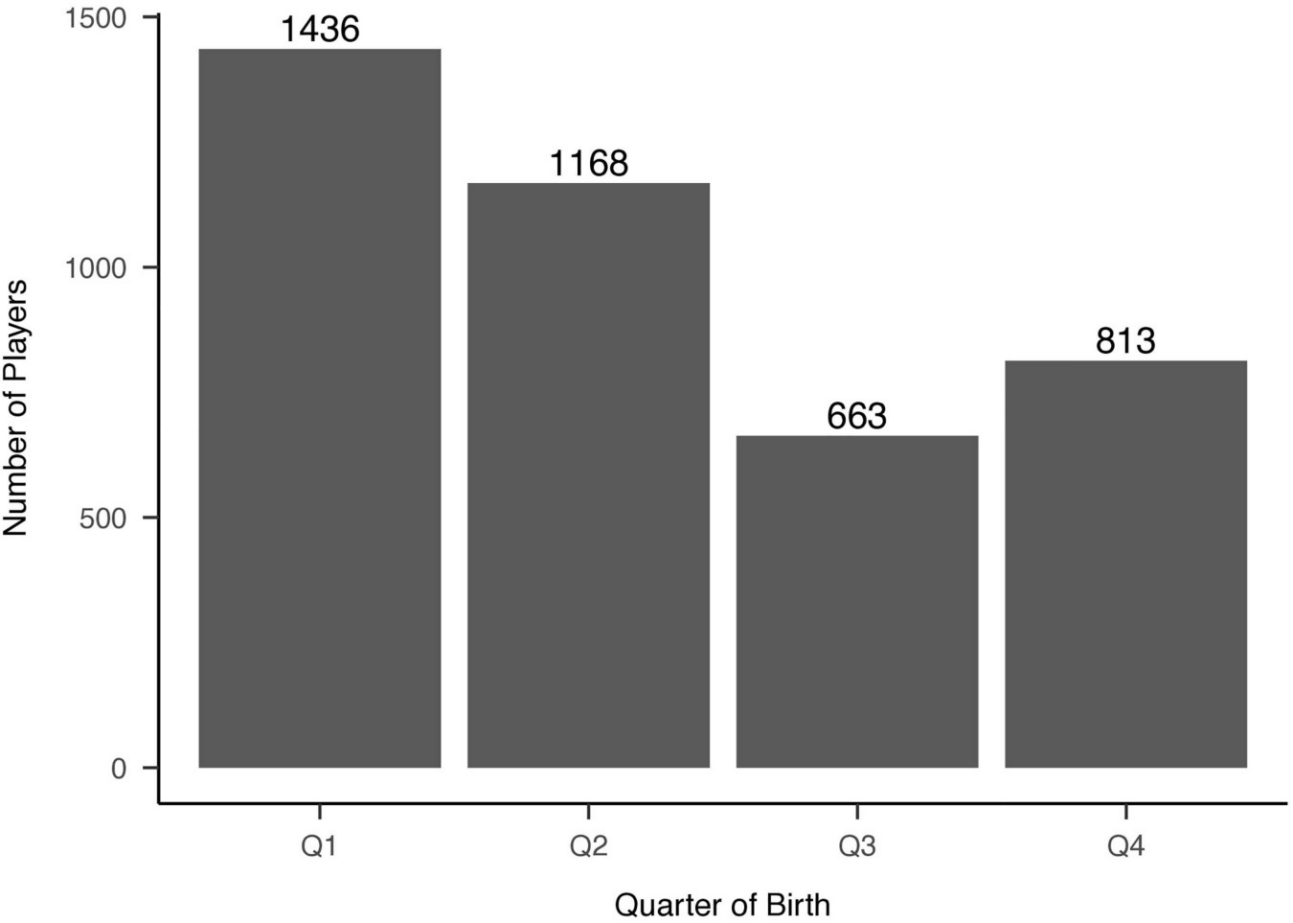
Distribution of players’ dates of birth quartiles (2005 onwards) by adjusted quartiles. Distribution of players’ birth quartiles (2005 onwards) using adjusted cut-offs, showing continued evidence of the relative age effect in recent NHL selections.

The distributions of birth quartiles using the adjusted quartile method can be found in Figure 6. By adjusting quartiles, Q3 athletes represented 14.65% of all selections compared with 20.03% previously, while Q4 athletes represented 20.78% compared with 15.40%, respectively. When using modified quartiles and examining athletes selected in 2005 and beyond (*n* = 4080), Q3 athletes represented 16.25% of all selections, while Q4 athletes represented 19.92% (Figure 7).

When examining time to entry and time to permanence using the post 2005 draft cohort with adjusted quartiles, Cox proportional hazards modeling found statistically significant effects of birth quartiles in both analyses (see Table 1 for comparison with standard quartile results). Evaluating time to entry showed that athletes selected from Q3 had a HR of 1.24 (95% CI = 1.08–1.42) relative to Q1, while Q4 exhibited nearly identical findings with a HR of 1.24 (95% CI = 1.08–1.40). When evaluating time to permanence using this cohort, Q3 athletes now demonstrated a HR of 1.61 (95% = 1.27–2.05) compared to Q1 athletes, while Q4 athletes demonstrated a HR of 1.24 (95% CI = 0.99–1.56).

**Table 1:** Summary of hazard ratios between standard and adjusted quartile analyses.

Birth quartile	Standard quartiles (1980–2024)		Adjusted quartiles (2005–2024)	
	Time to entry	Time to permanence	Time to entry	Time to permanence
Q1 (January–March)	Reference	Reference	Reference	Reference
Q2 (April–June)	0.94 (0.82–1.08)	1.03 (0.84–1.25)	1.04 (0.92–1.17)	1.23 (0.99–1.53)
Q3 (Jul–September)${}^{a}$	1.05 (0.89–1.25)	1.39 (1.10–1.75)**	1.24 (1.08–1.42)**	1.61 (1.27–2.05)***
Q4 (October–December)${}^{b}$	1.32 (1.15–1.52)***	1.25 (1.03–1.51)*	1.24 (1.08–1.40)**	1.24 (0.99–1.56)

Values show HR (95% CI). *p < 0.05, **p < 0.01, ***p < 0.001.

${}^{a}$Q3 in adjusted model = July 1–September 14.

${}^{b}$Q4 in adjusted model = September 15–December 31.

All models controlled for draft position, player position, BMI, nationality, and handedness.

## 4 Discussion

Relative age effects have been examined in ice hockey for over forty years, and despite significant research in this area, these effects persist. What seems clear is that an individual’s relative age is an influential factor in their long-term development, with similar findings seen in ice hockey using wide ranges of outcomes, including advanced analytics (12). Given that previous research has demonstrated that later born ice hockey athletes are equally, or more likely to be, successful at the NHL level (12, 34), and that disproportionate numbers of younger ice hockey players exist on junior hockey rosters (17, 35), RAEs most likely impact athletes prior to playing the highest levels of amateur hockey.

**Figure 8:**
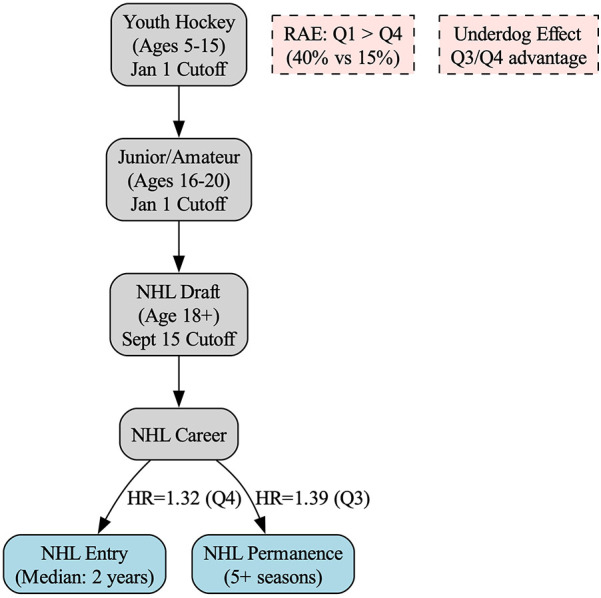
Conceptual framework of relative age effects across NHL development stages. This framework illustrates how relative age effects operate across developmental stages in ice hockey. The misalignment between youth hockey cut-off dates (January 1) and NHL draft eligibility (September 15) creates a dual disadvantage for Q3 athletes. Despite underrepresentation at draft, relatively younger players (Q3/Q4) who survive selection barriers demonstrate faster NHL entry and higher likelihood of achieving career permanence, supporting the underdog hypothesis.

When examining the two different outcomes with respect to RAE: time to entry and time to permanence, in both contexts, results revealed that relatively younger players were more likely to enter earlier than their relatively older peers, and that younger players were more likely to achieve permanence than older athletes in their selection cohort. These effects become even more pronounced in the adjusted quartiles for selections after 2005, where interestingly, Q3 athletes demonstrated the greatest probability of achieving permanence, even when controlling for draft selection. This finding is particularly notable given that these Q3 athletes face a “dual disadvantage” in North American hockey systems. Throughout their youth development, they compete as relatively younger players within the January 1 cut-off system, potentially facing age-related physical and competitive disadvantages. Then, when becoming eligible for NHL selection, they remain among the youngest in their draft cohort due to the September 15 cut-off. For instance, a player born in early September consistently competes as one of the younger athletes throughout youth hockey and continues to be among the youngest when draft-eligible. The notion that these dually disadvantaged athletes not only survive selection, but demonstrate superior career trajectories suggests remarkable resilience and skill development. Despite these findings, the number of adjusted Q3 athletes represent only 16.2% of selections, despite the time period representing just under 21% of the days in the calendar year. Similarly, using adjusted quartiles in the post 2005 cohort, Q4 athletes accounted for 19.9% of selections, while the time period accounted for 29.6% of the calendar year. As previously reported [see (17)], relatively younger athletes playing in leagues scouted by NHL teams may simply be more skilled than their relatively older peers in order to overcome some of the impacts of age. As illustrated in Figure 8, these Q3 athletes who overcome the dual disadvantage of misaligned cut-off dates demonstrate superior career trajectories, supporting the underdog hypothesis.

The exceptional performance of Q3 athletes in our study aligns with previous research suggesting these players possess compensatory skills. Despite being underrepresented in elite junior leagues that comprise the Canadian Major Junior Hockey League, including only 18.6%, 20.1%, and 16.7% of rosters in the Western Hockey League (WHL), Ontario Hockey League (OHL), and Quebec Maritimes Junior Hockey League (QMJHL) respectively, Q3 players who reach these levels demonstrate superior scoring ability compared to their relatively older peers (30). This pattern suggests that to overcome systematic age-related disadvantages throughout development, Q3 athletes must possess exceptional skill levels that ultimately translate into the faster NHL entry times and higher permanence rates observed in our data. Such findings provide strong empirical support for the underdog hypothesis, wherein the selection pressures faced by relatively younger players create a cohort of exceptionally talented survivors.

Time to permanence is likely impacted by a number of factors related to the athlete themselves, their playing pathway, and the team selecting the athlete. For example, an athlete selected at age 18 who has decided to play collegiate hockey in the National Collegiate Athletic Association (NCAA) may have a longer pathway to both enter the NHL and achieve permanence, given that they may choose to play collegiate hockey for four years. This pathway is notably different from an athlete playing in the Canadian Hockey League, who could only remain in amateur hockey until age 21, and until recently, could not play collegiate hockey in the NCAA. Despite these differing pathways, it is likely that athletes with the greatest ability would both enter and achieve permanence in the NHL quicker. This can be seen in both models presented in this paper, as the impact of relative age effects decreases when accounting for draft position (36).

This type of investigation offers a unique lens to examining the effects of age and player progression in the system. Given that relatively younger athletes appear to be equally or more successful then their relatively older peers, scouts and other stakeholders involved in selection may wish to adjust their approach to assessment, evaluation, and selection. It is possible that younger players who have successfully reached elite levels possess some type of compensatory factor (e.g., resilience, superior technical or tactical skill) that has allowed them to overcome the disadvantages of their birth timing. That said, more information would be valuable to learn about these relatively younger athletes in the sample, for instance, what systems they were in after they were drafted (e.g., how, where, and for how long athletes are called up, or sent down into the “farm” systems, etc.) as these can help shape the trajectory of the athlete’s career.

Importantly, there are several implications of this work. Perhaps most notably, this study focused on different elements of selection at the NHL level, but it is clear that the largest area in which RAE research should be considered is at the grassroots level. With Q3 and Q4 ice hockey athletes remaining a relatively small sample compared with their older peers at the level of NHL selection, efforts to better support younger athletes could have a range of benefits. Some of these solutions are not resource intensive, and include concepts such as bio-banding, which focuses on classifying athletes based on biological maturity rather than age alone (37, 38). Another method of accounting for better inclusion of relatively younger athletes (and thus more likely to be late maturing) is the use of “future teams” (39). Future teams are national level teams which select late maturing youth athletes (e.g., an U16 team that features athletes with physical profiles more similar to that of a U15 group), with the goal of retaining these athletes at the national level and providing them with access to better training, competition, and coaching (39). Future teams offer one form of athlete retention which may benefit athletes at the national level; however, this may be difficult to apply in some contexts (e.g., North American settings) where amateur teams are organized and administrated outside the control of a central national sport governing body.

### 4.1 Limitations

Despite what this investigation has to offer the sport and research community, it has limitations worth noting. First, our dataset only includes athletes who were actually drafted to the NHL, creating a selection bias that does not capture the full extent of relative age effects among all youth hockey players, particularly those who may have dropped out of competitive hockey before reaching draft eligibility. Second, while we controlled for anthropometric measures (height, weight, BMI) at draft, these variables represent physical size rather than biological maturation status. Without direct measures of maturation such as peak height velocity or skeletal age (5), we cannot make definitive claims about maturational differences between birth quartiles. Indeed, research in other sports has shown that some relatively younger athletes may be early maturing, potentially compensating for chronological age disadvantages (19, 29). Future research incorporating actual maturation assessments would provide more complete understanding of how biological development interacts with relative age effects.

A third limitation is that our analysis does not account for variations in the development systems and league structures across different countries, which may influence how relative age effects manifest and impact player development trajectories before reaching the NHL draft. Finally, while we examined time to entry and permanence, we did not analyze specific performance metrics or playing styles that might differ between relatively older and younger players, which could provide further insights into how these athletes adapt and succeed despite age-related disadvantages.

Additionally, we acknowledge that this analysis provides an incomplete picture of athlete performance and development. Performance-based outcomes such as scoring statistics, time on ice, and advanced metrics (e.g., Corsi numbers, expected goals) could provide valuable insights into how relative age affects on-ice performance at multiple career stages. As noted by Baker, Johnston, and Wattie (40), this means we capture only those who “survived” the development system, missing the potentially larger population of relatively younger athletes who were released from competitive hockey earlier. Finally, this dataset contained only men’s hockey players, limiting our ability to generalize findings across genders and missing the opportunity to compare relative age effects between men’s and women’s professional hockey development pathways.

### 4.2 Future directions

Future work could benefit from incorporating longitudinal tracking of players from youth hockey through to professional leagues, analyzing not just career outcomes but also the developmental pathways, playing styles, and specific skills that relatively younger players develop to overcome systemic disadvantages in the selection process.

To address the selection bias inherent in examining only drafted players, future studies can implement population-based cohort designs that capture complete age cohorts from youth hockey entry through draft eligibility, documenting attrition patterns and exit points for relatively younger athletes. This would require partnerships with national hockey federations to access comprehensive registration data across developmental stages.

International comparative research examining how structural variations in development systems moderate relative age effects could provide crucial insights. Different countries employ varying age cut-off dates, league structures, and player development models’systematic comparison of these approaches would identify which organizational features best support relatively younger athletes’ development and retention.

The integration of performance analytics represents another critical avenue. Future research should incorporate advanced metrics (e.g., expected goals, Corsi, zone entries, passing efficiency) and player tracking data to examine whether successful relatively younger players exhibit distinct performance profiles or compensatory advantages. Time-series analyses of these metrics could reveal how playing styles evolve across career stages and whether early disadvantages translate into later advantages in specific performance domains.

Finally, extending this analytical framework to women’s professional hockey would address current gender limitations and test the generalizability of findings. Given potential differences in maturation rates, competitive structures, and career opportunities between men’s and women’s hockey, comparative analyses could yield important theoretical and practical insights for developing more equitable talent development systems across all levels of the sport.

## 5 Conclusion

Relative age effects in ice hockey have been extensively studied, and while new research continues to extend these effects, the overall pattern of results has been highly consistent over 40 years. Until systemic level change is adequately implemented at the grassroots level, relatively younger athletes will remain underrepresented. Athletes who are relatively younger (whether true Q4, or adjusted quartile Q3/Q4 as we discuss) will likely succeed due to having already survived the systemic barriers they encountered. Overall, using new outcomes and modified cut-offs, we identified findings that further support the extensive research on this topic indicating that relatively younger athletes are underrepresented, but are equally or more successful than their relatively older peers. Future research should prioritize how grassroot and amateur ice hockey systems can allow for better developmental opportunities for relatively younger and less biologically mature athletes.

## Data Availability

The original contributions presented in the study are included in the article/Supplementary Material, further inquiries can be directed to the corresponding author/s.
